# Supplementary material for: Thiamine transporter 2 and Janus kinase 2 inhibitor, fedratinib suppresses thermogenic activation of human neck area-derived adipocytes
Source: Front Endocrinol (Lausanne). 2026 Feb 10;17:1694542. doi: 10.3389/fendo.2026.1694542 (PMC12929129; doi:10.3389/fendo.2026.1694542)
Supplement: Supplementary file 1 [file DataSheet1.pdf]

Supplementary Material

Thiamine transporter 2 and Janus kinase 2 inhibitor, fedratinib, suppresses thermogenic activation of human neck area-derived adipocytes

Gyath Karadsheh[1,2], Emília Kovács[1,3], Rahaf Alrifai[1,2], Mizuki Seo[1,2], Ferenc Győry[4], Renáta Csatári-Kovács[5], Éva Csősz[5], Szilárd Póliska[6], László Fésüs[1], Rini Arianti[1,*,†], Endre Kristóf[1,*,†]

*** Correspondence:** Endre Kristóf: kristof.endre@med.unideb.hu; Rini Arianti: ariantirini@med.unideb.hu

1 Supplementary Figures and Tables

1.1 Supplementary Figures

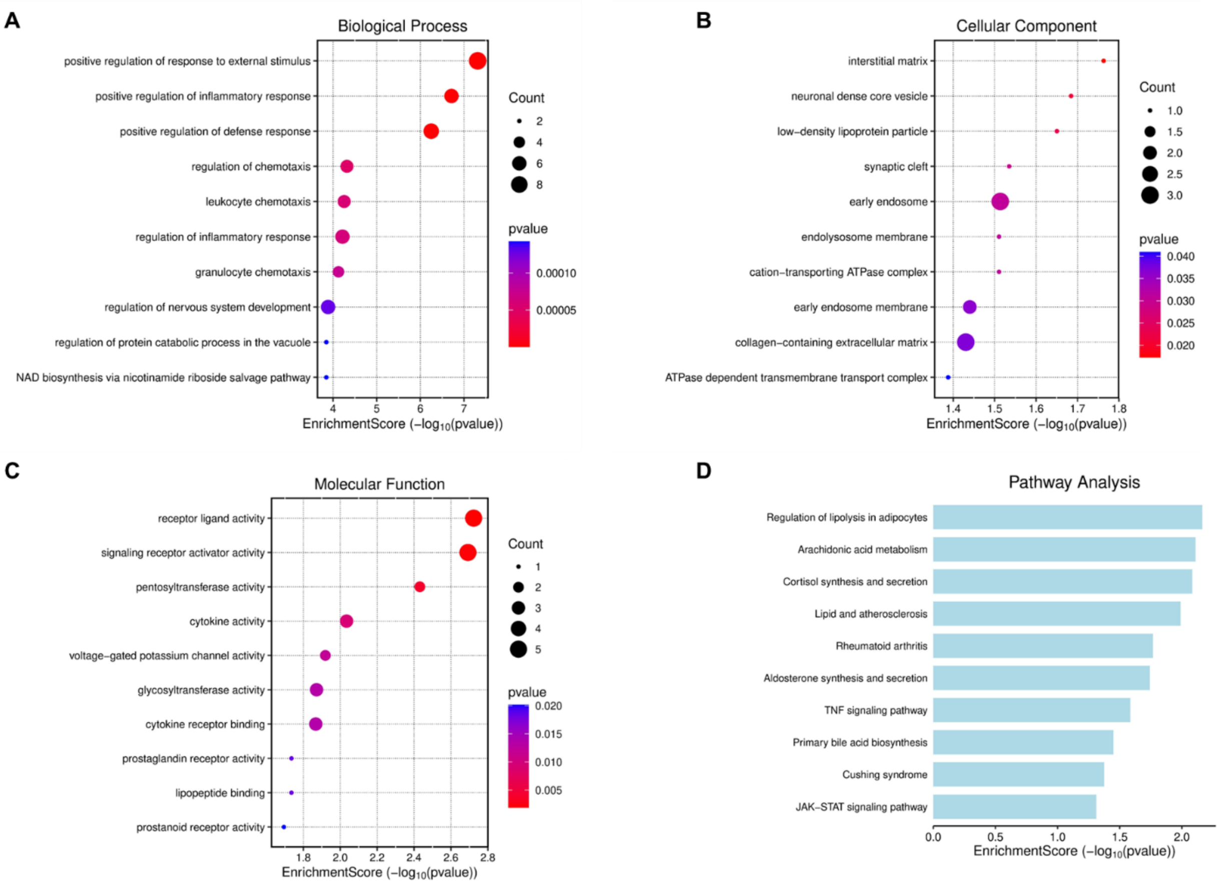

Supplementary Figure 1. Gene Ontology enrichment in subcutaneous (SC)-derived adipocytes (A) Biological processes, (B) cellular components, (C) molecular functions, and (D) pathway analysis of the differentially expressed genes that were suppressed by fedratinib during dibutyryl-cAMP-

stimulated thermogenesis, enriched in SC-derived adipocytes [Data cited from www.bioinformatics.com.cn/SRplot].

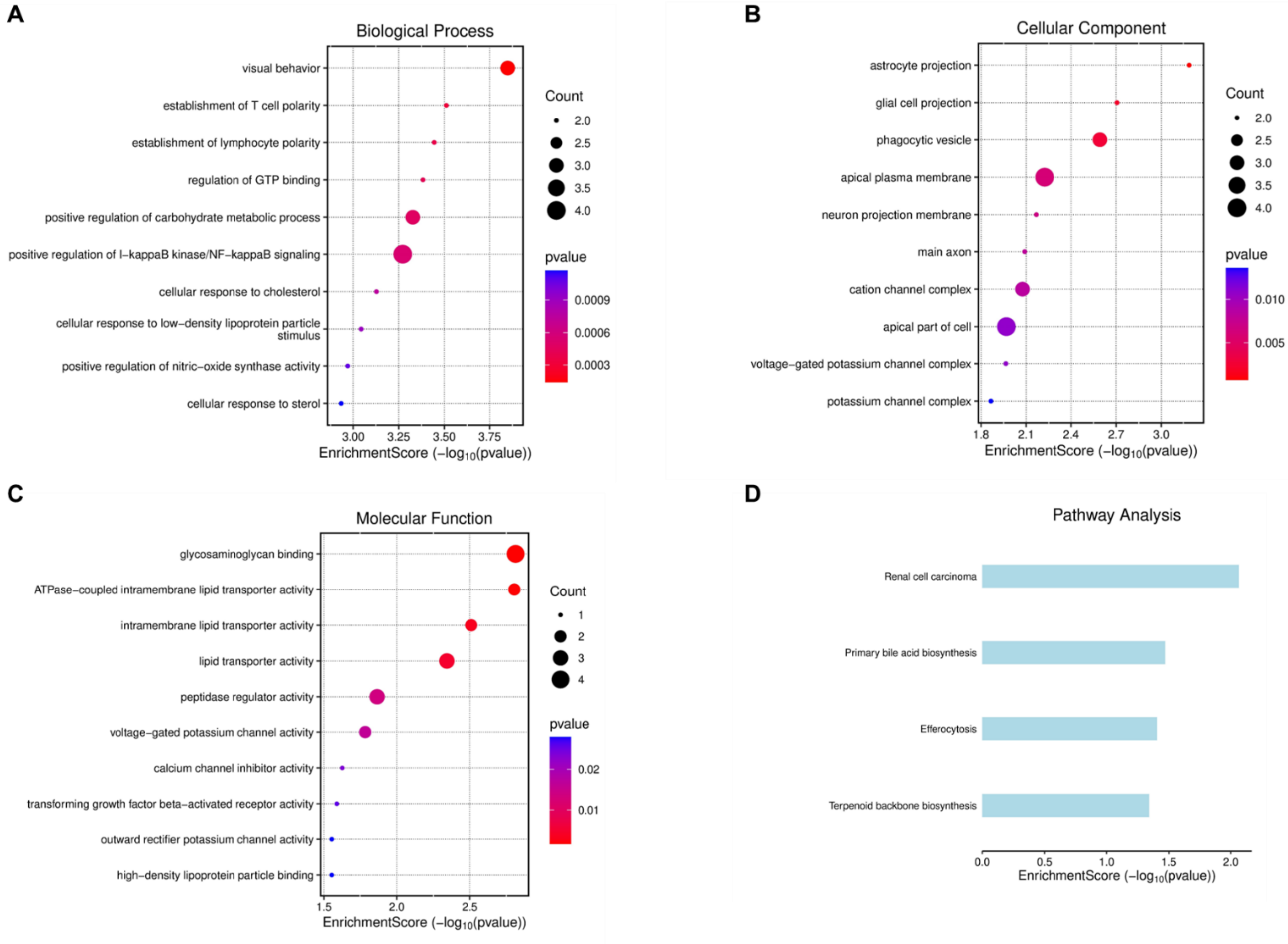

Supplementary Figure 2. Gene Ontology enrichment in deep neck (DN)-derived adipocytes (A) Biological processes, (B) cellular components, (C) molecular functions, and (D) pathway analysis of the differentially expressed genes that were suppressed by fedratinib during dibutyryl-cAMP-stimulated thermogenesis, enriched in DN-derived adipocytes [Data was cited from www.bioinformatics.com.cn/SRplot].

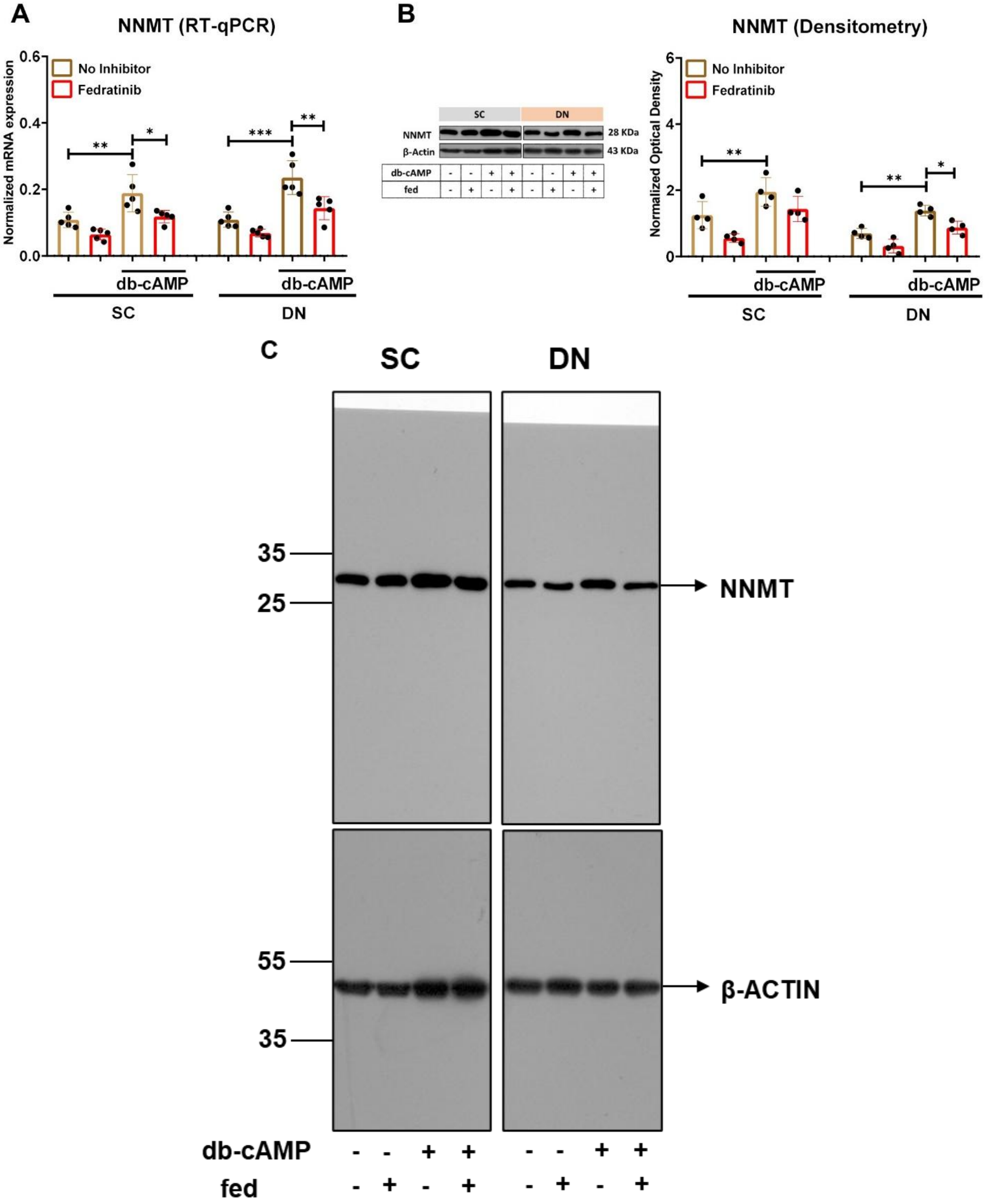

Supplementary Figure 3. Effect of thiamine inhibition on the NNMT in subcutaneous (SC) and deep neck (DN)-derived adipocytes after 10 h of dibutyryl (db)-cAMP induced thermogenic activation. (A) The mRNA expression of *NNMT* analyzed by RT-qPCR. (B) Protein expression of NNMT detected by immunoblotting. (C) Uncropped images presented with molecular weight ladders

for panel B. n=5 for RT-qPCR and n=4 for immunoblotting. Statistical analysis was performed by one-way ANOVA followed by Tukey's *post hoc* test, *p<0.05, **p<0.01, and ***p<0.001.

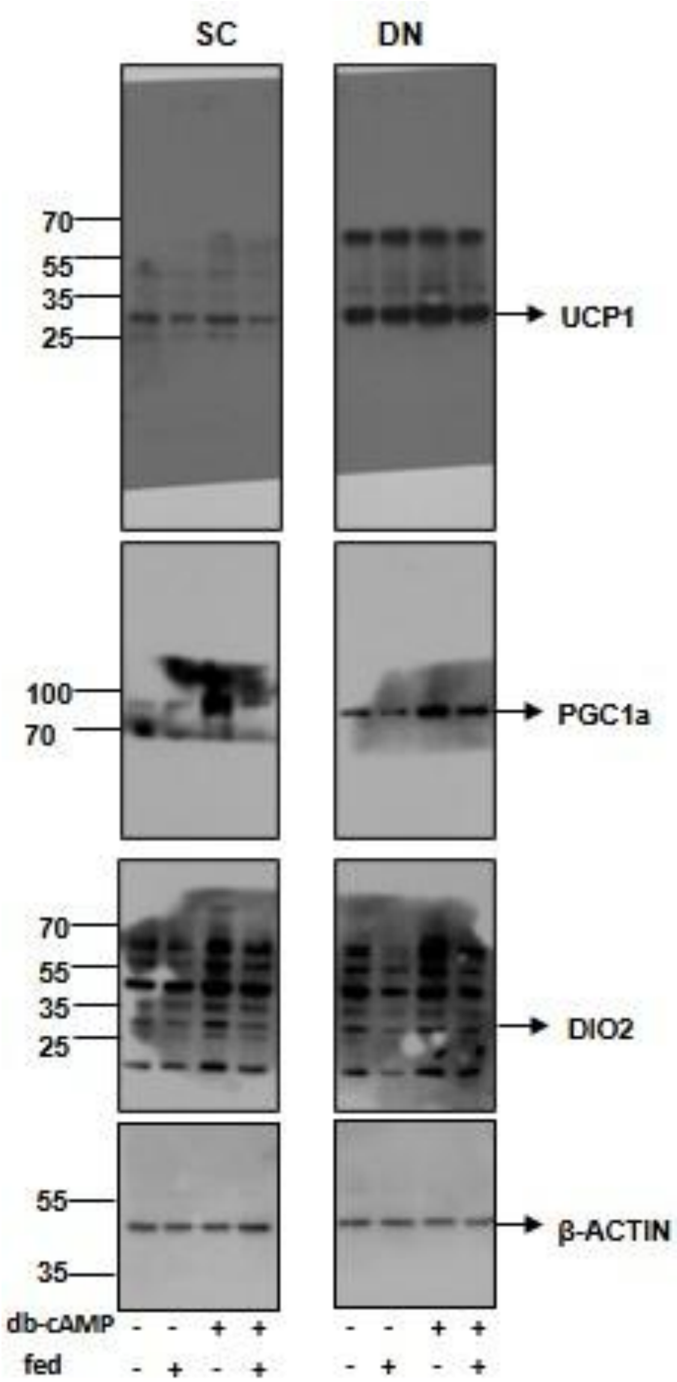

Supplementary Figure 4. Uncropped images presented with molecular weight ladders for Figure 2B. β-actin was used as endogenous control. Detailed information regarding the antibodies and working dilution are displayed in Supplementary Table 2.

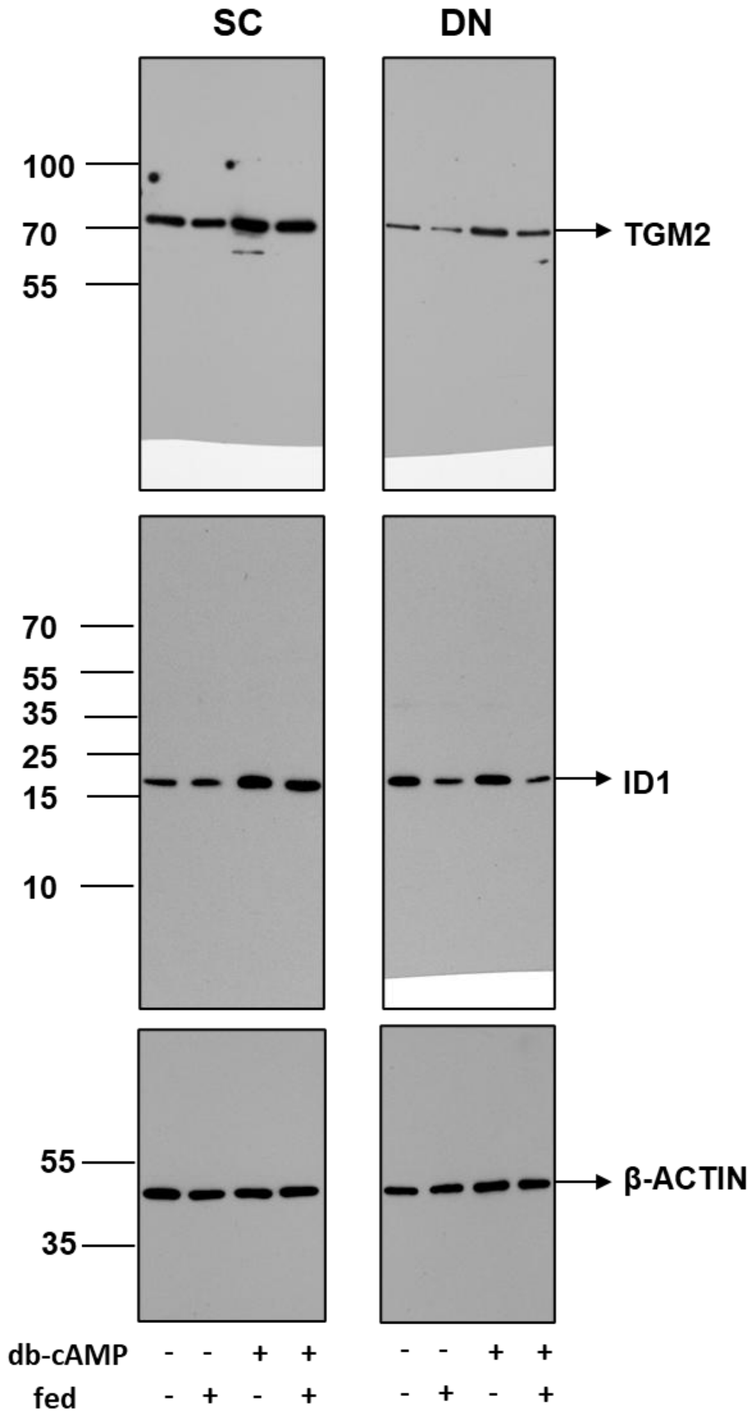

Supplementary Figure 5. Uncropped images presented with molecular weight ladders for Figure 4B. β-actin was used as endogenous control. Detailed information regarding the antibodies and working dilution are displayed in Supplementary Table 2.

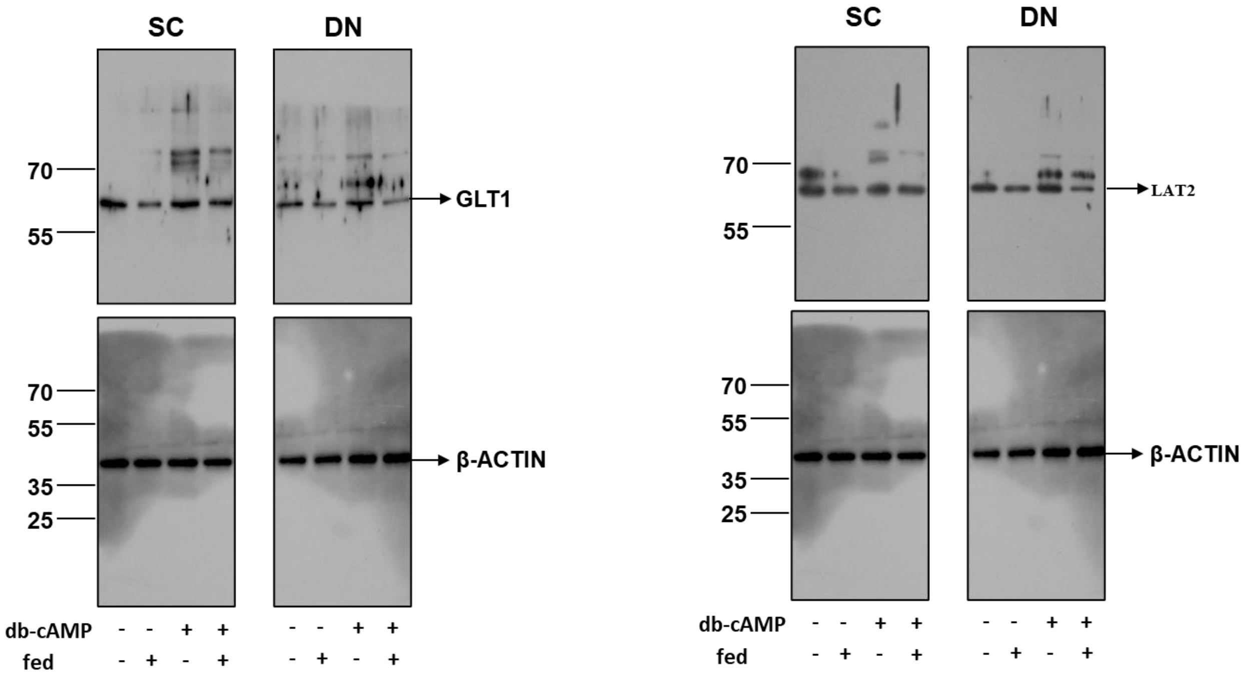

Supplementary Figure 6. Uncropped images presented with molecular weight ladders for Figure 5D (left panel) and 5G (right panel). β-actin was used as an endogenous control. Detailed information regarding the antibodies and working dilution is displayed in Supplementary Table 2**.**

A

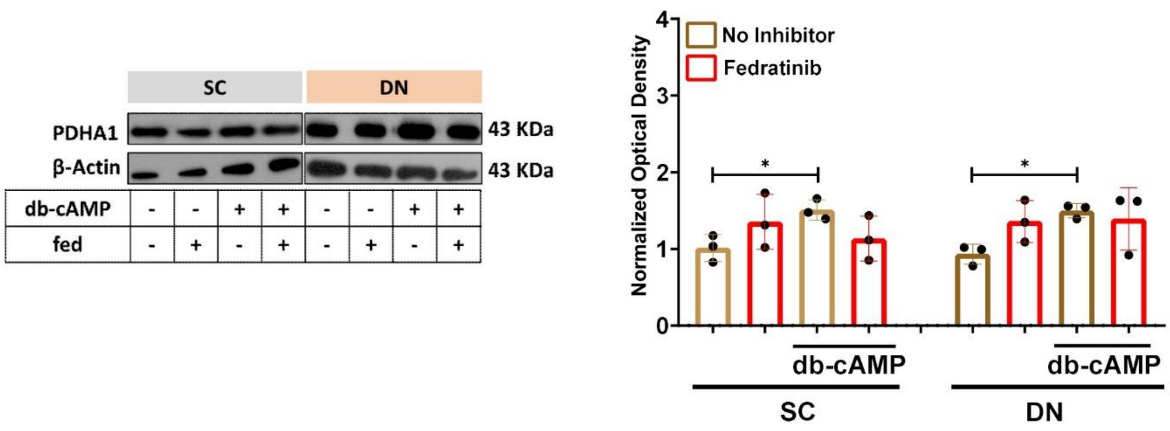

B

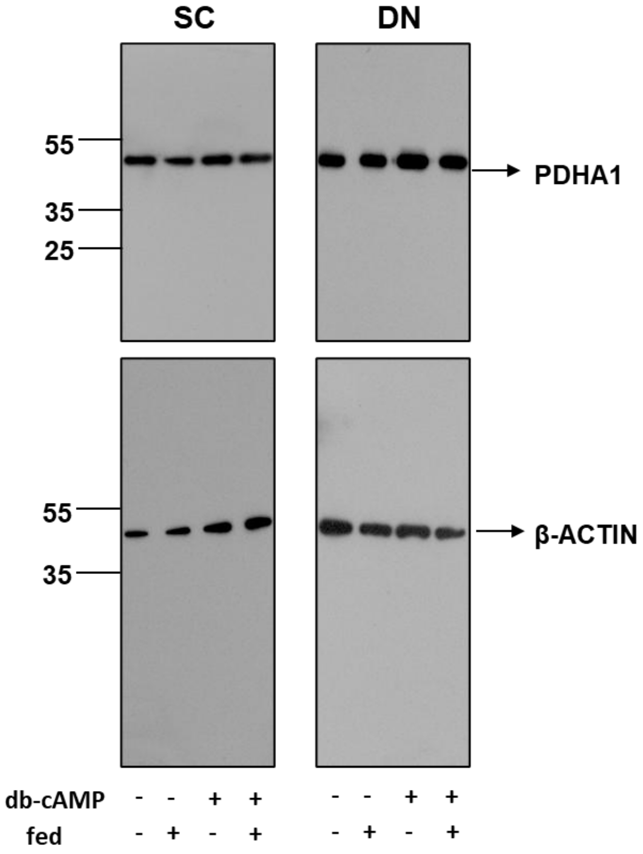

Supplementary Figure 7. Effect of thiamine inhibition on PDHA1 protein expression in subcutaneous (SC) and deep neck (DN)-derived adipocytes after 10 h of dibutyryl (db)-cAMP induced thermogenic activation. (A) Protein expression of PDHA1 detected by immunoblotting, n=3. (B) Uncropped images presented with molecular weight ladders for panel A. Statistical analysis was performed by one-way ANOVA followed by Tukey's *post hoc* test, *$p<0.05$.

1.2 Supplementary Tables

Supplementary Table 1. Gene primers and probes

GENES	ASSAY ID
ACTB	Hs01060665_g1
DIO2	Hs00399438_m1
GAPDH	Hs99999905_m1
ID1	Hs00179727_m1
NNMT	Hs00196287_m1
PGC1A	Hs01075227_m1
SLC1A2	Hs001102423_m1
SLC7A8	Hs00794796_m1
TGM2	Hs001096681_m1
UCP1	Hs00222453_m1

Supplementary Table 2. Antibodies used in immunoblotting

Antibody	Company	Catalog Number	Dilution
UCP1	R&D Systems, Minneapolis, MN, USA	MAB6158	1:750
PGC1a	Santa Cruz Biotechnology, Inc., Dallas, TX, USA	SC-517380	1:1000
DIO2	Thermo Fisher Scientific, Waltham, MA, USA	PA5-49631	1:2000
TGM2	ABCAM, Cambridge, UK	AB109200	1:2000
NNMT	Cell Signaling Technology, Danvers, MA, USA	24912S	1:1000
ID1	Novus Biologicals, CO, USA	NBP2-66897	1:1000

GLT1	Thermo Fisher Scientific, Waltham, MA, USA	PA5-117492	1:1000
LAT2	Thermo Fisher Scientific, Rockville, MD, USA	TA500515	1:2000
ACTIN	Sigma-Aldrich, Munich, Germany	A2066	1:10000
PDHA1	Invitrogen, Waltham, MA, USA	459400	1:1000
HRP-conjugated goat anti-rabbit IgG	Advansta, San Jose, CA, USA	R-05072-500	1:5000
HRP-conjugated goat anti-mouse IgG	Advansta, San Jose, CA, USA	R-05071-500	1:5000

Supplementary Table 3. Differentially expressed genes (DEGs) from comparison of db-cAMP vs db-cAMP and fedratinib in subcutaneous adipocytes; n=3

Symbol	Gene Name	Log_2 Fold Change	Adjusted P value
TGM2	transglutaminase 2	2.15	2.33E-20
PLA2G2A	phospholipase A2 group IIA	1.62	9.73E-09
KCNK3	potassium two pore domain channel subfamily K member 3	1.16	0.000546816
CHRDL2	chordin like 2	1.15	0.001034331
STEAP4	STEAP4 metalloreductase	1.15	0.001150183
NNMT	nicotinamide N-methyltransferase	1.14	9.60E-05
LBP	lipopolysaccharide binding protein	1.13	0.00084268
NTRK1	neurotrophic receptor tyrosine kinase 1	1.08	0.002846505
MGAT3	beta-1,4-mannosyl-glycoprotein 4-beta-N-acetylglucosaminyltransferase	1.07	0.002234289
ADAMTS15	ADAM metallopeptidase with thrombospondin type 1 motif 15	1.06	0.001034331
PHYHIP	phytanoyl-CoA 2-hydroxylase interacting protein	1.01	0.008220822
TYMP	thymidine phosphorylase	1.00	0.002141746
EPSTI1	epithelial stromal interaction 1	1.00	0.011890579
STAT4	signal transducer and activator of transcription 4	0.99	0.010003999
SPOCD1	SPOC domain containing 1	0.98	0.01556629
ID1	inhibitor of DNA binding 1, HLH protein	0.97	0.015646124
CCL2	C-C motif chemokine ligand 2	0.95	0.019714157
SEMA6B	semaphorin 6B	0.94	0.023618465
LDLR	low density lipoprotein receptor	0.93	0.000850014
RIPOR3	RIPOR family member 3	0.92	0.028817648
HSD3B7	hydroxy-delta-5-steroid dehydrogenase, 3 beta- and steroid delta-isomerase 7	0.92	0.000850014
IL15	interleukin 15	0.92	0.015026787
SCG2	secretogranin II	0.91	0.0345513
FER1L6	fer-1 like family member 6	0.91	0.0345513
APOL3	apolipoprotein L3	0.90	0.027995467
KRT7	keratin 7	0.89	0.041862404
CCDC177	coiled-coil domain containing 177	0.88	0.0345513
C6orf132	chromosome 6 open reading frame 132	0.88	0.010502331
PARP9	poly(ADP-ribose) polymerase family member 9	0.87	0.01556629

DIO2	iodothyronine deiodinase 2	0.86	0.010318771
EGFLAM	EGF like, fibronectin type III and laminin G domains	0.85	0.0345513
SPRY1	sprouty RTK signaling antagonist 1	0.84	0.008975406
CDC42EP4	CDC42 effector protein 4	0.84	0.001034331
LOC730101	uncharacterized LOC730101	0.83	0.041862404
NREP	neuronal regeneration related protein	0.82	0.007592763
RIPOR2	RHO family interacting cell polarization regulator 2	0.81	0.020487659
LURAP1L	leucine rich adaptor protein 1 like	0.78	0.01556629
PTGS1	prostaglandin-endoperoxide synthase 1	0.76	0.04404196
ABCC9	ATP binding cassette subfamily C member 9	0.76	0.035580322
PTGER3	prostaglandin E receptor 3	0.72	0.023618465
ATF4	activating transcription factor 4	-0.72	0.010533499
MT2A	metallothionein 2A	-0.72	0.047898625
KIF3A	kinesin family member 3A	-0.75	0.015646124
MT1X	metallothionein 1X	-0.77	0.026258133
ETV5	ETS variant transcription factor 5	-0.77	0.010318771
MTHFD2	methylenetetrahydrofolate dehydrogenase (NADP+ dependent) 2, methenyltetrahydrofolate cyclohydrolase	-0.83	0.015646124
SLC38A1	solute carrier family 38 member 1	-0.88	0.000466027
FOSB	FosB proto-oncogene, AP-1 transcription factor subunit	-0.88	0.01556629
BIRC3	baculoviral IAP repeat containing 3	-0.91	0.0345513
HS3ST2	heparan sulfate-glucosamine 3-sulfotransferase 2	-0.91	0.0345513
ADM2	adrenomedullin 2	-0.92	0.020487659
CYP2S1	cytochrome P450 family 2 subfamily S member 1	-0.96	0.008220822
GPAT3	glycerol-3-phosphate acyltransferase 3	-0.97	0.01556629
APOLD1	apolipoprotein L domain containing 1	-1.17	3.61E-06
RRAD	RRAD, Ras related glycolysis inhibitor and calcium channel regulator	-1.19	0.00076711

Supplementary Table 4. Differentially expressed genes (DEGs) from comparison of db-cAMP vs db-cAMP and fedratinib in deep neck adipocytes; n=3

Symbol	Gene Name	Log_2 Fold Change	Adjusted P value
RIPOR2	RHO family interacting cell polarization regulator 2	1.38	1.20E-06
SPRY1	sprouty RTK signaling antagonist 1	1.37	2.93E-06
IL1RL1	interleukin 1 receptor like 1	1.34	0.000173883
KCNQ3	potassium voltage-gated channel subfamily Q member 3	1.28	0.000179194
STON1	stonin 1	1.28	0.000739228
LYVE1	lymphatic vessel endothelial hyaluronan receptor 1	1.24	0.001772437
ATP8B4	ATPase phospholipid transporting 8B4 (putative)	1.23	0.001032291
ARHGAP28	Rho GTPase activating protein 28	1.22	0.00017712
LURAP1L	leucine rich adaptor protein 1 like	1.21	2.93E-06
MYPN	myopalladin	1.21	0.001109771
NNMT	nicotinamide N-methyltransferase	1.17	0.000179194
DIRAS3	DIRAS family GTPase 3	1.09	0.015930663
CCIN	calicin	1.08	0.015362152
LXN	latexin	1.06	0.001032291
APOL3	apolipoprotein L3	1.04	0.018921816
HSD3B7	hydroxy-delta-5-steroid dehydrogenase, 3 beta- and steroid delta-isomerase 7	1.03	3.87E-06
BMPR1B	bone morphogenetic protein receptor type 1B	1.01	0.003292177
IGFN1	immunoglobulin like and fibronectin type III domain containing 1	1.01	0.039038322
HGF	hepatocyte growth factor	1.01	0.031949474
CDC42EP4	CDC42 effector protein 4	1.00	2.93E-06
PIK3IP1	phosphoinositide-3-kinase interacting protein 1	1.00	0.038695784
LINC01605	long intergenic non-protein coding RNA 1605	1.00	0.038173261
LMO7	LIM domain 7	0.97	0.001065127
ZNF436	zinc finger protein 436	0.95	0.010478386
SLC1A2	solute carrier family 1 member 2	0.95	0.006485879
HRH1	histamine receptor H1	0.95	0.045849513
TRAF3IP2	TRAF3 interacting protein 2	0.94	0.003540079
SERPINB2	serpin family B member 2	0.94	0.004616777
PHYHIP	phytanoyl-CoA 2-hydroxylase interacting protein	0.92	0.032505943
TYMP	thymidine phosphorylase	0.89	0.021333805
NOD1	nucleotide binding oligomerization domain containing 1	0.89	0.015930663

HIF1A	hypoxia inducible factor 1 subunit alpha	0.88	0.001215782
ABI3BP	ABI family member 3 binding protein	0.87	0.006193246
KCNK2	potassium two pore domain channel subfamily K member 2	0.86	4.97E-05
JADE2	jade family PHD finger 2	0.86	0.004821908
ABCA1	ATP binding cassette subfamily A member 1	0.86	0.015930663
CLEC2B	C-type lectin domain family 2 member B	0.83	0.0074486
ADAMTS5	ADAM metallopeptidase with thrombospondin type 1 motif 5	0.83	0.003656475
ZNF423	zinc finger protein 423	0.76	0.032505943
MCUB	mitochondrial calcium uniporter dominant negative subunit beta	0.69	0.016657911
HMGCS1	3-hydroxy-3-methylglutaryl-CoA synthase 1	0.67	0.001618602
ATF4	activating transcription factor 4	-0.58	0.032057653
NR4A1	nuclear receptor subfamily 4 group A member 1	-0.82	0.014001714
BAIAP2	BAR/IMD domain containing adaptor protein 2	-0.85	0.04573663
H2AC6	H2A clustered histone 6	-0.90	0.004791084
ASNS	asparagine synthetase (glutamine-hydrolyzing)	-0.95	0.0199421
H4C8	H4 clustered histone 8	-0.99	0.043864342
ARC	activity regulated cytoskeleton associated protein	-1.05	0.018687134
